# Application of two-sample Mendelian randomization method to assess the causal relationship between rheumatoid arthritis and osteoporotic fracture

**DOI:** 10.3389/fmed.2024.1388968

**Published:** 2024-05-10

**Authors:** Cai Zhenyu, Chang Le, Zeng Shiyong, Lin Jinding, Liu Mingzhong, Tang Haifeng, Zeng Rongdong

**Affiliations:** ^1^Department of Orthopedics, Quanzhou First Hospital Affiliated of Fujian Medical University, Quanzhou, Fujian, China; ^2^Department of Clinical Medicine, Quanzhou Medical College, Quanzhou, Fujian, China; ^3^Medical Research Center of Quanzhou Medical College, Quanzhou, Fujian, China

**Keywords:** rheumatoid arthritis, inflammatory disease, osteoporosis, osteoporotic fracture, Mendelian randomization

## Abstract

**Background:**

The association between rheumatoid arthritis (RA) and osteoporotic fracture has garnered considerable attention; however, the causal relationships between diseases remain uncertain. Therefore, this study employed Mendelian randomization (MR) analysis to investigate the causal effects of RA on osteoporotic fracture.

**Methods:**

The summary data for RA and osteoporotic fracture were extracted from the genome-wide association studies (GWAS) catalog and the Finn Biobank database. The database provides information about diseased and health control subjects. We searched the database for the following conditions: RA, osteoporosis (OP), and osteoporotic fractures. Entries were published by investigating centers, which had established definitions and diagnostic criteria. We downloaded and processed the data to obtain the single-nucleotide polymorphisms (SNPs) strongly associated with RA, OP, and osteoporotic fracture. RA genetic associations were obtained from the GWAS catalog, including 1961 cases and 454,387 controls. The osteoporosis of the GWAS catalog involved 991 cases and 455,357 controls, and the data of the Finn Biobank involved 8,017 cases and 391,037 controls. Genetic associations for osteoporotic fracture were taken from the Finn Biobank of 1822 cases and 311,210 controls. Independent SNPs that are significantly associated with meeting the criteria of *p* < 5 × 10–8, r2 < 0.001, and kb = 10,000 were selected for MR analysis. The inverse variance-weighted (IVW) method along with other MR methods was employed for analysis, while sensitivity analyses were conducted to assess reliability and stability.

**Results:**

The results provided strong evidence that RA was causally and positively associated with osteoporosis from the GWAS catalog (OR = 1.16590; 95% CI: 1.04067–1.30619; *p* = 0.00811) and the Finn Biobank database (OR = 1.07314; 95% CI: 1.03455–1.11317; *p* = 0.00016). Moreover, a positive causal relationship was detected between RA and osteoporotic fracture (OR = 1.10132; 95% CI: 1.00506–1.20680; *p* = 0.03863). The results were robust according to sensitivity tests.

**Conclusion:**

This study showed positive causal relationships between RA and osteoporotic fracture. These results should be considered in further studies and public health measures on osteoporosis prevention strategies.

## Introduction

Osteoporosis (OP) is a metabolic bone disorder characterized by reduced bone mass and deterioration of bone microstructure, resulting in diminished skeletal strength or heightened susceptibility to fragility fractures (1). Population aging has become a global phenomenon (2), and the United Nations predicts that by 2050, approximately a quarter of the population in most regions will be above the age of 60 years. Approximately 200 million women worldwide are affected by osteoporosis, with one-third of women and one-fifth of men aged 50 years or above experiencing an osteoporotic fracture (3).

Inflammation is the response of the body to various pathogens and tissue damage, involving the activation of cells from both innate and adaptive immune responses and the production of cytokines, such as tumor necrosis factor-alpha (TNF-α), interleukins, chemokines, and interferon (4). Bone loss represents a clinical manifestation of chronic inflammatory diseases and can be readily observed in rheumatoid arthritis (RA) (5). Recent research has put forth the theory of inflammation-related osteopenia, which posits that RA can induce osteoporotic fracture through inflammatory cytokine activity (6). However, the use of glucocorticoids in patients with RA makes it difficult to assess whether RA or glucocorticoids cause osteoporosis. The precise causal relationship between RA and osteoporotic fracture still requires further elucidation.

The advent of genome-wide association analysis (GWAS) has revolutionized the field of human genetics and complex diseases, greatly enhancing our understanding of genetic mechanisms (7). Mendelian randomization (MR), an approach that utilizes GWAS data as instrumental variables (IVs), allows for the exploration of causal effects between exposures and outcomes (8). Single-nucleotide polymorphisms (SNPs) are DNA sequence polymorphisms caused by variation of a single nucleotide at the genomic level. MR is a research method that uses SNPs strongly correlated with exposure factors as IVs to infer causal effects between exposure factors and study outcomes. A causal relationship in MR analysis is the existence of exposure that could cause an outcome. In other words, exposure is the cause and effect is the outcome. If exposure is present, protective factors are attributed to a lower probability of the outcome, whereas risk factors indicate a higher probability of the outcome. By virtue of genotypes being present before disease onset, remaining constant throughout life, and largely independent from postnatal lifestyle or environmental factors, MR can effectively minimize confounding and overcome limitations associated with traditional observational studies (9). Therefore, MR represents a viable approach to analyze the causal relationships between RA and osteoporotic fracture. The phenomenon of horizontal pleiotropy arises when a single SNP exerts its influence on the outcome not only through its association with exposure but also indirectly or directly via other SNPs. This may potentially violate one of the fundamental assumptions underlying Mendelian randomization, which posits that SNPs can solely impact outcomes through their effects on exposure. The leave-one-out method is employed to evaluate whether a single SNP exerts an excessive impact on the overall MR estimate. The procedure involves sequentially removing one SNP at a time and subsequently re-estimating the MR using the remaining SNPs. This iterative process is repeated for each individual SNP. If there is a significant change in the overall estimate after removing a particular SNP, it suggests that this specific SNP may have an undue influence on the MR estimate or could be biased. In this study, we conducted a two-sample MR analysis using a large sample GWAS meta-analysis to investigate the causal relationships between RA and osteoporotic fracture.

## Methods

### Study design and data sources

**Figure 1:**
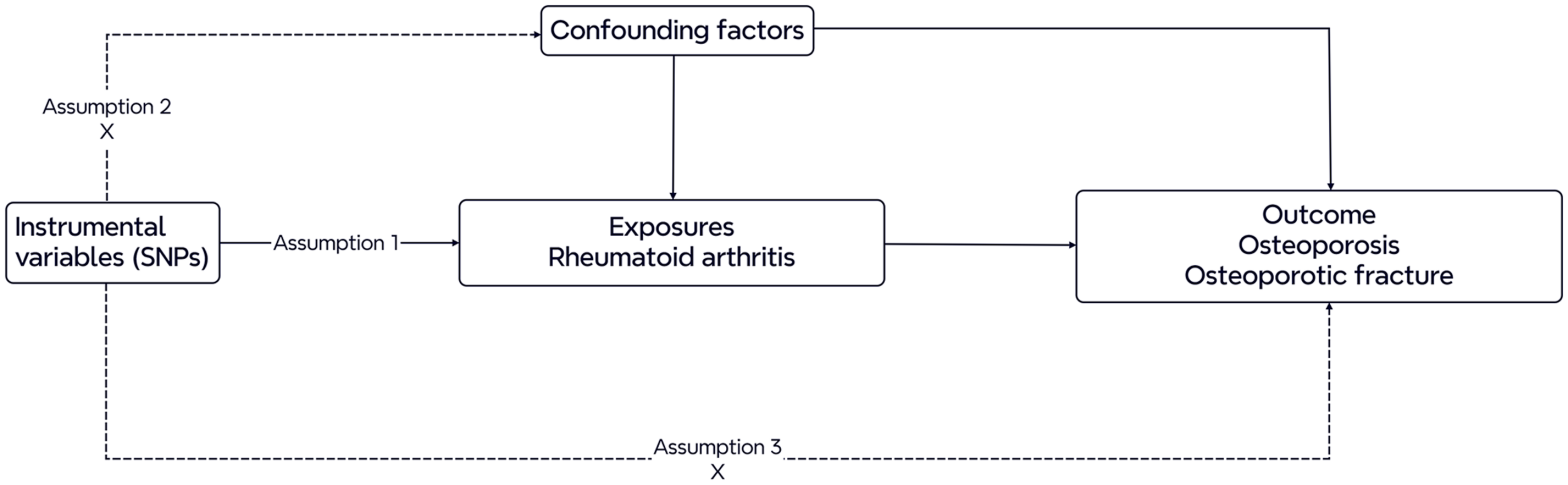
Diagram of the Mendelian randomization study. Three assumptions are required. First, the genetic variants should be closely related to the exposures. Second, the genetic variants are supposed to be independent of confounding factors. Third, the effects of the genetic variants on outcomes are only mediated by the exposures.

Two-sample Mendelian randomization was utilized in this study to investigate the causal associations of RA with bone osteoporosis and osteoporotic fracture. RA was categorized as the exposure, and osteoporosis and osteoporotic fracture were considered outcomes. Figure 1 shows the diagram of the study design for this MR analysis. Three main assumptions are basic conditions in the MR analysis (7): First, the instrumental variables (IVs) should be closely related to the exposures; second, the IVs should be independent of confounding factors; and finally, the IVs of exposures should only influence the outcome (no horizontal pleiotropy). The genome-wide SNPs were regarded as IVs at a significant level (*p* < 5 × 10–8). To avoid the bias caused by linkage disequilibrium (LD), we performed a clumping procedure with *r*^2^ < 0.001 or physical distance between them was within 10,000 kb utilizing data from the European ancestry-based 1,000 Genomes Project (10). Furthermore, we calculated *F* statistics for each exposure and F statistics >10 were considered suggestive of adequate instrument strength (11). Finally, we utilized a program that harmonized the discordant alleles and palindromic SNPs to generate a summary set of the same effect allele between exposures and outcomes. Finally, the selected SNPs were investigated in subsequent MR analysis.

The data used in the MR analyses originated from the GWAS catalog and the Finn Biobank. RA genetic associations were obtained from the GWAS catalog including 1961 cases and 454,387 controls. The osteoporosis of the GWAS catalog involved 991 cases and 455,357 controls, and the data of the Finn Biobank involved 8,017 cases and 391,037 controls. Genetic associations for osteoporotic fracture were taken from the Finn Biobank of 1822 cases and 311,210 controls. All genetic data utilized in this study used populations of European descent. Table 1 provides details of the data sources used and the demographic profiles of RA and outcomes (osteoporosis and osteoporotic fracture). Ethical approval and consent of the participants included in the previously published GWAS summary data have already been obtained. The database is freely accessible for investigators, and therefore, ethical approval for the current study is not required.

**Table 1:** Data sources used in this study.

Exposure or outcomes	Sample size (cases/controls)	Ancestry	Consortia	GWAS ID
Rheumatoid arthritis	1961/454387	European	GWAS	GCST90044540
Osteoporosis	991/455357	European	GWAS	GCST90044600
Osteoporosis	8017/391037	European	Finn Biobank	R10_M13_OSTEOPOROSIS
Osteoporotic fracture	1822/311210	European	Finn Biobank	R10_OSTEOPOROSIS_FRACTURE_FG

### Statistical analyses

To assess the causal effects of RA on outcomes, five different MR methods were employed in this MR analysis. The IVW method was our primary MR analysis, which provides unbiased estimates of the causal effect as long as all genetic variants are valid instruments, and the results showed no heterogeneity or pleiotropy (12). The IVW method calculates the exposure–outcome effect for each SNP using the Wald ratio method and conducts a weighted linear regression with a forced intercept of zero. This method is known for its higher estimation accuracy and test power when the IVs satisfy three assumptions. To account for potential interference from unknown and unmeasurable confounders, MR-Egger regression (MR-Egger) was performed. Additionally, we used the weighted median method, simple mode method, and weighted mode method, which provide consistent estimates of causality even when up to 50% of the information comes from invalid IVs. In order to enhance the reliability of this MR analysis, MR-Egger, weighted median, simple mode, and weighted mode methods were used as supplementary analyses. According to the type of variables in this MR analysis, the results were reported as odds ratios (ORs) with 95% confidence intervals (CIs). *p*-values below 0.05 for the IVW method indicate a significant causal effect of exposures on the outcome, and the supplementary methods were consistent with IVW. All statistical analyses were performed using the two-sample MR (version 0.5.8) package in R software version 4.3.2. The results were considered to be statistically significant when the *p*-value was <0.05.

### Sensitivity analysis

To assess heterogeneity, we utilized Cochran’s Q statistic and a *p*-value greater than 0.05 reflects no heterogeneity. A random-effect model would be adopted in the subsequent analyses when heterogeneity existed; otherwise, a fixed-effect model would be adopted (13). The intercept term of MR-Egger regression was conducted because a *p*-value above 0.05 indicates no pleiotropic effect of the IVs. Moreover, a significant horizontal pleiotropic effect of individual SNPs inducing bias was evaluated with “leave-one-out” sensitivity test (14).

## Results

### The extracted SNPs for Mendelian randomization analyses

In this two-sample MR study, RA was taken as exposure, while osteoporosis and osteoporotic fracture were taken as outcomes. We obtained five SNPs (rs10065637, rs2476601, rs35139284, rs6910879, and rs9275602) associated with exposure, and each kind of exposure was included in this study. The mean values of F statistics for selected IVs of exposure and outcomes was 183.92. All of the *F* statistics were larger than 10, demonstrating that the selected IVs were strong enough to decrease any potential bias of the causal analyses.

### Causal relationship of RA on osteoporosis

**Figure 2:**
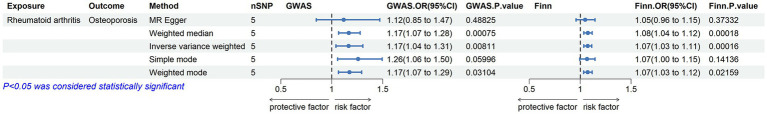
Associations of genetic liability to RA with risk of osteoporosis in the GWAS catalog (UKB) and the Finn Biobank study.

**Figure 3:**
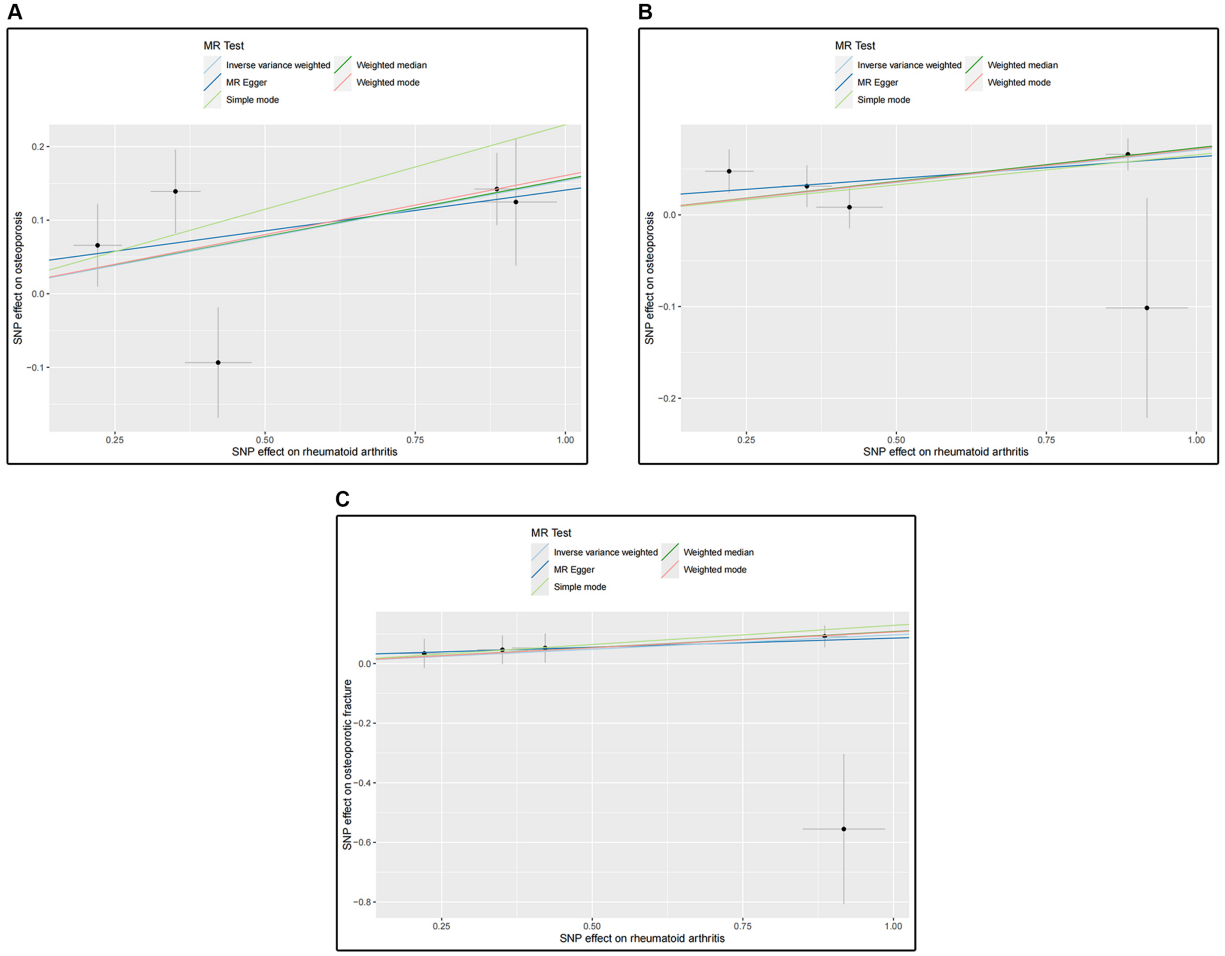
**(A)** Scatter plot for analysis of RA and osteoporosis in the GWAS catalog; **(B)** Scatter plot for analysis of RA and osteoporosis in the Finn Biobank; **(C)** Scatter plot for analysis of RA and osteoporotic fracture in the Finn Biobank.

The MR results for the causal effects of RA on osteoporosis obtained from the GWAS catalog database are listed in Figures 2, 3A. Genetic liability to RA was associated with an increased risk of osteoporosis as analyzed by the IVW method (OR = 1.16590; 95% CI: 1.04067–1.30619; *p* = 0.00811). Similar results could also be acquired by weighted median (OR = 1.16803; 95% CI: 1.06714–1.27847; *p* = 0.00075) and weighted mode (OR = 1.17418; 95% CI: 1.06617–1.29312; *p* = 0.03104). Heterogeneity has not been observed based on Cochran’s *Q* tests (*Q* value in MR-Egger = 6.84501; *p* = 0.07701), and no pleiotropy was detected in this study (MR-Egger intercept = 0.03015; *p* = 0.75291) (Table 2). The results were stable and reliable because of the assessment of the “leave-one-out” sensitivity, which revealed the individual SNPs could not change the causal relationship (Supplementary Figure S1A).

**Table 2:** MR sensitivity analyses of between exposures and outcome.

Exposure	Outcomes	Heterogeneity tests		Directional horizontal pleiotropy test	
		methods	Cochran’s Q (*P*)	MR-Egger intercept	Pleiotropy *p*-value
RA	Osteoporosis (GWAS)	MR-Egger	6.84501 (0.07701)	0.030145	0.75291
		Inverse variance-weighted	7.11648 (0.12986)		
	Osteoporosis (Finn)	MR-Egger	4.29157 (0.23165)	0.01607	0.60059
		Inverse variance-weighted	4.77844 (0.31080)		
	Osteoporotic fracture	MR-Egger	6.53658 (0.08823)	0.02399	0.75781
		Inverse variance-weighted	6.78512 (0.14769)		

The MR results shown in Figures 2, 3B determined the positive causal effects of RA on osteoporosis obtained from the Finn Biobank. The results of MR analysis were IVW method (OR = 1.07314; 95% CI: 1.03455–1.11317; *p* = 0.00016), weighted median method (OR = 1.06712; 95% CI: 1.03548–1.11753; *p* = 0.00016), and weighted mode method (OR = 1.07435; 95% CI: 1.03387–1.11641; *p* = 0.02159), respectively. There was no heterogeneity between the individual SNPs (Cochran’s *Q* value in MR-Egger = 4.29157; *p* = 0.23165), and the results of MR-Egger regression suggested that horizontal pleiotropy was not existing to bias the causation relationship (MR-Egger intercept = 0.01607; *p* = 0.60059) (Table 2). The “leave-one-out” analysis could not detect a single SNP (Supplementary Figure S1B).

### Causal relationship of RA on osteoporotic fracture

**Figure 4:**
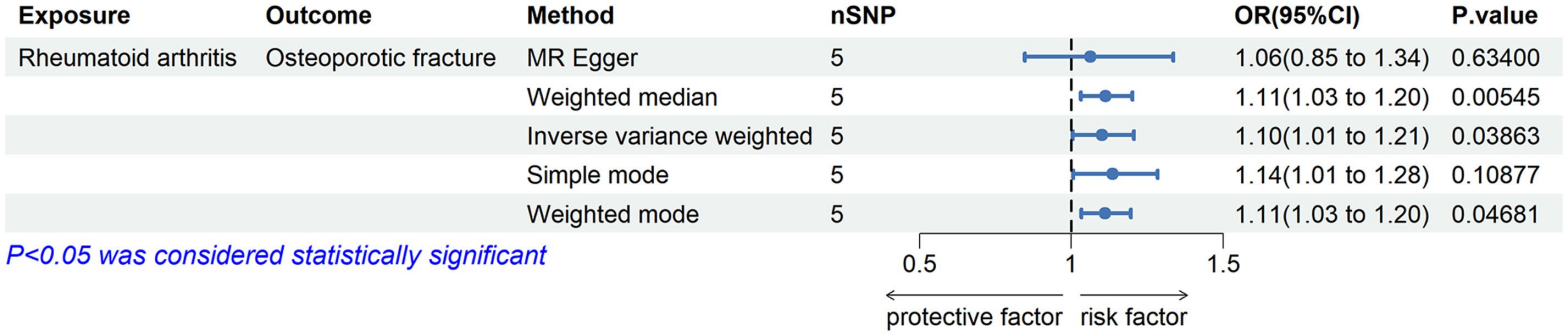
Associations of genetic liability to RA with risk of osteoporotic fracture in the Finn Biobank study.

We evaluated the causal effects of RA on osteoporotic fracture (Figures 3C, 4). In the primary IVW analyses, RA showed an increased risk of osteoporotic fracture (OR = 1.10132; 95% CI: 1.00506–1.20680; *p* = 0.03863). As the supplementary analyses, the results of MR analysis were weighted median method (OR = 1.11335; 95% CI: 1.03216–1.20094; *p* = 0.00545) and weighted mode method (OR = 1.11182; 95% CI: 1.03342–1.19616; *p* = 0.04681), respectively. Furthermore, according to the results, Cochran’s *Q* tests (Cochran’s *Q* value in MR-Egger = 6.53658; *p* = 0.08823) reflected that there was no heterogeneity between the individual SNPs. No pleiotropy (MR-Egger intercept = 0.02399; *p* = 0.75781) was found in the results according to the MR-Egger regression (Table 2). The “leave-one-out” analysis demonstrated that the identified causal relationship between RA and osteoporotic fracture was not altered by a single SNP (Supplementary Figure S1C).

All results of the scatter plots for causal relationships between RA and osteoporosis are shown in Figure 3. The “leave-one-out” analysis plots are shown in Supplementary Figure S1, funnel plots, and forest plots are presented in Supplementary Figures S2, S3.

## Discussion

In the present study, we used a two-sample Mendelian randomization analysis to determine whether RA is causally related to osteoporosis and osteoporotic fracture. Through the GWAS catalog and the Finn Biobank data, our results suggested the existence of causal relationships between RA and osteoporosis and osteoporotic fracture.

The main characteristics of osteoporotic fracture include a decrease in bone mineral density and an increase in fragility fracture events, resulting in a significant burden on the healthcare system. Therefore, osteoporosis and osteoporotic fracture are considered one of the most notable diseases (15). Although recent research studies indicated that inflammatory diseases have a remarkable impact leading to the increase in osteoporotic fracture risk, which is detrimental to bone health, the potential mechanism of inflammatory diseases on osteoporotic fracture is complex (16).

Patients with inflammatory diseases are exposed to long-term chronic inflammatory factors. This could result in a reduction in bone density, joint destruction, subsequent osteoporotic fractures, and increased risk of mortality (17). The balance of the activity between the osteoblasts and osteoclasts is a critical factor in bone health. The inflammatory cytokines contributed to osteoporosis with increased bone resorption and decreased bone formation by breaking the balance of osteoblastogenesis and osteoclastogenesis (18).

Currently, there are numerous theories regarding the mechanism by which RA increases the risk of osteoporotic fracture, primarily involving an inflammatory state, cytokine release, and autoantibodies. The role of autoimmunity in osteoporosis was discussed in previously published studies. Such as anti-citrullinated autoantibodies and anti-carbamylated proteins were demonstrated to be associated with decreased bone mass in patients with RA (19, 20). Chronic inflammation is a clinical manifestation of RA. The inflammatory response in RA is predominantly driven by heightened secretion of cytokines, such as TNF-α, IL-6, and IL-1, which can directly or indirectly activate osteoclasts and result in bone loss. Additionally, inflammatory cytokines can impede osteoblast differentiation (21). Research has also demonstrated that IL-34 can facilitate the differentiation of inflammatory monocytes infiltrating joints into osteoclasts and expedite bone resorption (22). Other studies have identified RANKL as one of the key cytokines involved in both local and systemic bone loss pathogenesis in RA patients. In individuals with RA, RANKL mainly originates from CD4+ and CD28-T cells. Under these circumstances, RANKL can enhance osteoclast activity while inhibiting osteoblast development (23, 24). Dkk-1 serves as another major regulator of bone remodeling within Wnt signaling pathway inhibition; its elevated levels are associated with an increased risk of osteoporotic fracture and bone erosion among RA patients (25).

According to previous research, osteoporosis is a common condition in subjects with RA. A study conducted on an Asian population revealed that approximately 33.6% of individuals with RA had osteoporosis, which was significantly higher than the control group (12.7%) (26). Furthermore, Kim et al. (27) concluded that the incidence of osteoporosis in RA patients (22.1%) was approximately twice as high as that in healthy individuals (11.4%). However, due to their condition requirements, RA patients require treatment with higher doses and longer durations of glucocorticoids and disease-modifying antirheumatic drugs (DMARDs) for disease improvement. It is well established that glucocorticoids and DMARDs have side effects leading to osteoporosis development. Raterman et al. (28) discovered that methotrexate can directly impact bone metabolism in RA patients by inhibiting osteoblast activity, resulting in decreased bone mineral density (BMD) and increased fracture risk. Guler-Yuksel et al. (29) observed local and systemic osteoporosis development prior to diagnosis among some RA patients, where reduced bone mineral density may primarily be associated with the use of DMARDs or glucocorticoid therapy. In individuals with inflammatory diseases without utilizing glucocorticoids, BMD is primarily influenced by demographic factors such as age and gender (29, 30). These studies suggest potential confounding factors such as glucocorticoids, DMARDs, age, and gender influencing decline in BMD during clinical observational studies among patients with RA. In a recent case–control study in the Spanish population, Gomez-Vaquero et al. observed a high incidence of clinical fragility fractures in postmenopausal women with rheumatoid arthritis (31). The main risk factors were having a previous fracture, RA disease activity and disability, and the cumulative dose of glucocorticoids. Furthermore, in the same cohort, Guañabens et al. showed that the risk of vertebral fracture in RA is still high in recent years when compared with the general population (32). Once more, the key determinants of fracture risk are age, glucocorticoids, and falls.

Mendelian randomization study is a research method utilized to investigate the causal relationship between diseases. In MR analysis, genetic variation is determined at conception and remains unaffected by external factors, making it akin to a randomized controlled trial. Due to the ongoing health concern regarding the association between RA and osteoporosis, scholars have recently employed the Mendelian randomization method to investigate the genetic-level causal relationship between these two diseases. However, there is inconsistency in the conclusions drawn from these studies. Liu et al. (33) were the first to utilize bidirectional Mendelian randomization and found no current evidence supporting a causal relationship between RA and osteoporosis. Similar findings were also observed by Song et al. (34), which included European and Asian populations but failed to provide evidence for causation. Conversely, Deng et al. (35) demonstrated positive support for a causal relationship between RA and osteoporosis specifically within the Japanese population.

This study employed a two-sample MR analysis to genetically evaluate the causal effect of RA on osteoporotic fracture. We utilized more updated data from the GWAS catalog than that used in the published articles, thereby enhancing the scientific rigor of our study. Our findings provide robust evidence supporting a causal relationship between RA and osteoporosis. To ensure the reliability of these results, we further employed the latest data from the Finn Biobank database, which consistently confirmed a stable causal relationship between the two diseases. However, there are certain limitations in this study. First, subgroup analysis was not conducted based on different types of osteoporotic fractures, such as primary osteoporosis fractures, postmenopausal osteoporosis fractures, or drug-induced osteoporosis fractures. Second, the study only utilized the GWAS catalog and the Finn Biobank database without incorporating any other GWAS databases, potentially introducing bias. Finally, the sample population for this study consisted solely of individuals of European descent. Considering that allele frequencies may introduce bias in genetic studies when applied to ethnically diverse populations, thus limiting its interpretation and application in other ethnic groups.

## Conclusion

In conclusion, this study confirms the existence of a positive causal relationship between RA and osteoporotic fracture. These findings should be taken into consideration in future research endeavors as well as when formulating public health measures and strategies for preventing osteoporosis.

## Data availability statement

The original contributions presented in the study are included in the article/Supplementary material, further inquiries can be directed to the corresponding author.

## Author contributions

CZ: Writing – review & editing, Writing – original draft. CL: Writing – review & editing, Writing – original draft. ZS: Writing – original draft. LJ: Writing – original draft. LM: Writing – original draft. TH: Writing – original draft. ZR: Writing – review & editing, Writing – original draft.
