# Supplementary figures and images for: Application of two-sample Mendelian randomization method to assess the causal relationship between rheumatoid arthritis and osteoporotic fracture
Source: Front Med (Lausanne). 2024 May 10;11:1388968. doi: 10.3389/fmed.2024.1388968 (PMC11116583; doi:10.3389/fmed.2024.1388968)

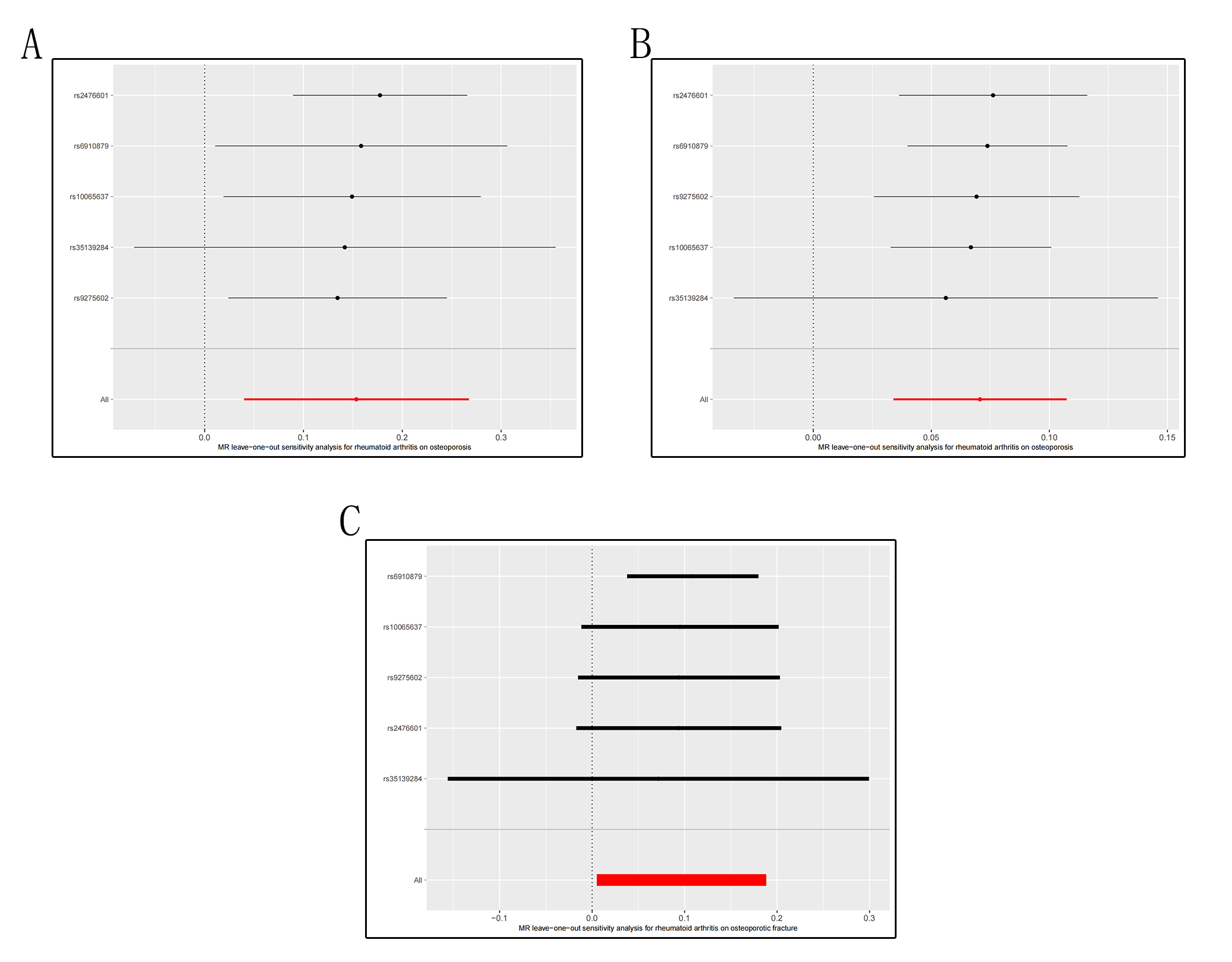

Supplement: Supplementary file 1 [file Image_1.JPEG]

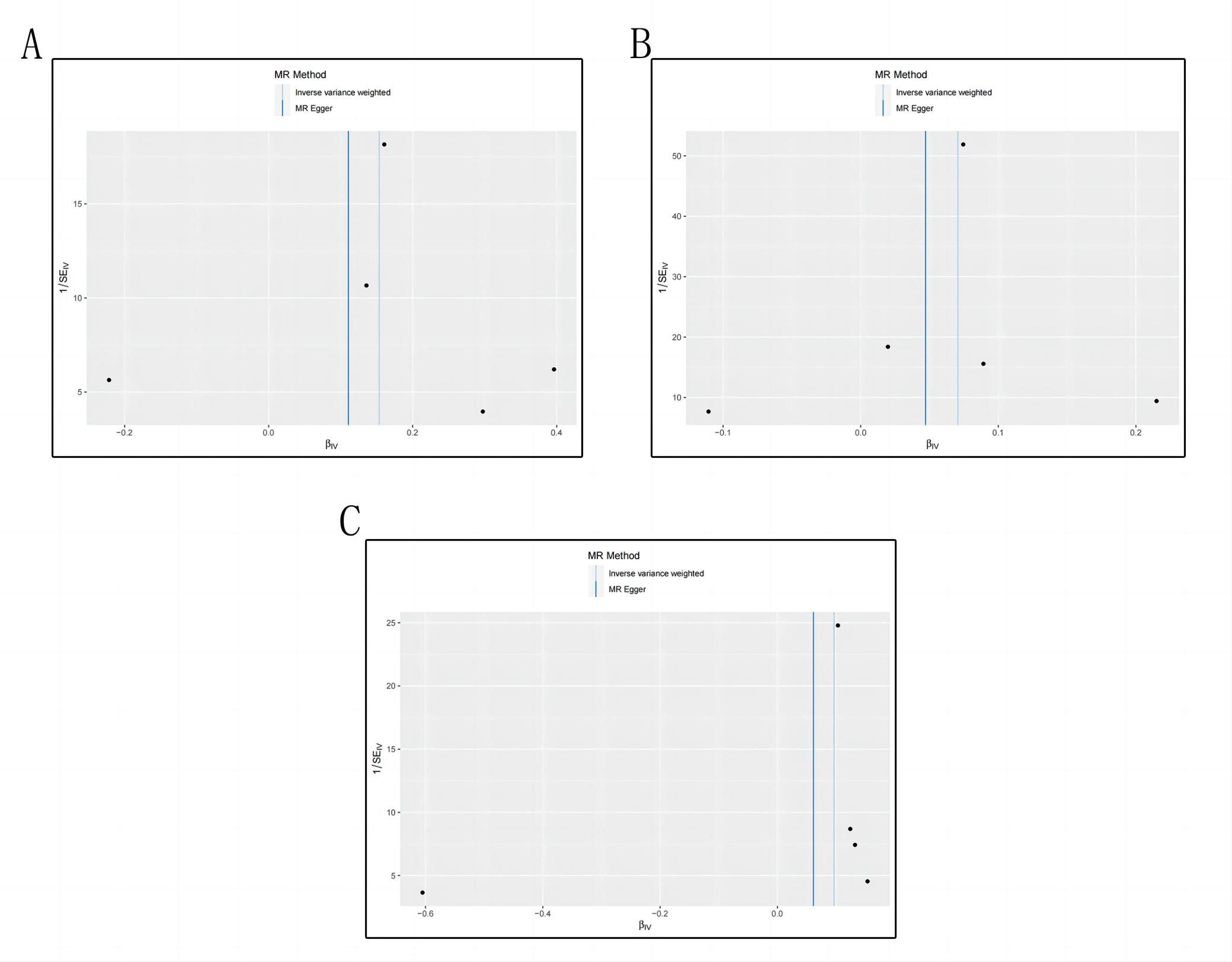

Supplement: Supplementary file 2 [file Image_2.JPEG]

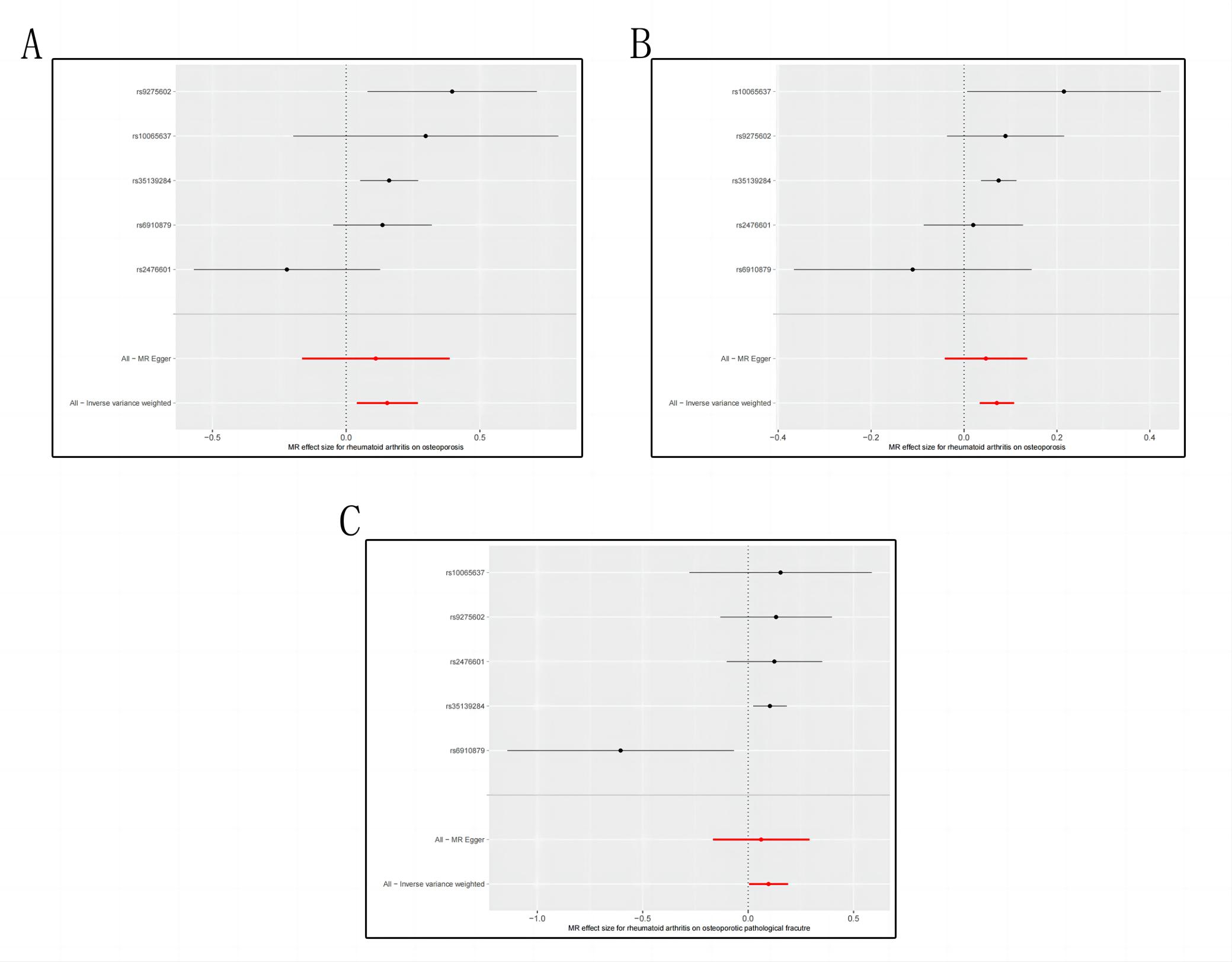

Supplement: Supplementary file 3 [file Image_3.JPEG]
